# Factors influencing sexual and reproductive health among adolescents in Lao PDR

**DOI:** 10.1080/16549716.2020.1791426

**Published:** 2020-08-03

**Authors:** Khampheng Phongluxa, Ghislaine Langeslag, Tej Ram Jat, Sengchanh Kounnavong, Mariam A. Khan, Dirk R. Essink

**Affiliations:** a Lao Tropical and Public Health Institute, Ministry of Health, Vientiane, Lao PDR; b Athena Institute, Faculty of Earth and Life Sciences, Vrije Universiteit Van Amsterdam, Amsterdam, The Netherlands; c United Nations Population Fund (UNFPA), Vientiane, Lao PDR

**Keywords:** LEARN: Sexual Reproductive Health, ANC and Nutrition, Contraception, autonomy, gender inequality, gender roles, gender-based violence

## Abstract

**Background:**

Adolescents are particularly vulnerable to poor sexual and reproductive health outcomes. In addition, Lao PDR has the highest teenage pregnancy rate in southeast Asia and a high maternal mortality ratio.

**Objective:**

This study aimed to provide a comprehensive exploration of factors that influence SRH knowledge, attitudes, and practices of adolescents in Bokeo Province, Lao PDR.

**Method:**

Data from the Adolescent Girl Situation Analysis cross-sectional study, collected in 2018 using a mixed-method approach with 837 adolescents aged 10–19, and key informant interviews, were analysed. Regression analyses were used to identify predictors of modern contraception knowledge, autonomy, gender-based violence, sexual activity, and contraception use. This was complemented with qualitative thematic content analysis.

**Results:**

Adolescents living in two rural districts had lower sexual and reproductive health knowledge compared to urban district residents. Findings showed misconceptions about the birth control pill, a belief that sex education is important, but that the current teaching quality is problematic. There was a strong positive association between knowledge and autonomy. In the two rural districts, residents were more likely to lack autonomy. Marriage was described as an autonomous decision, yet 40.4% lacked autonomy regarding marriage. Among sexually active adolescents, 35.2% used contraception. Boys and girls were said to be equal, yet education access and gender roles favoured boys. Additionally, violence was more justified by husbands against their wives.

**Conclusion:**

The study helps to understand the views and perceptions of adolescents and key informants on gender equality and gender-based violence. Three main areas require more effort and greater investment to improve adolescent sexual and reproductive health: knowledge and use of contraceptives, gender inequality, and autonomy. There is poor knowledge of contraceptive methods, indicating a need to further integrate comprehensive sexual education, introduced in primary school, and to increase investment in training and monitoring teachers.

## Background

The right to sexual and reproductive health (SRH) encompasses the ability to have a safe, responsible, and satisfying sex life, as well as having the capability to reproduce, and to decide if, when, with whom, and how to do so [1]. There are many inequalities in realising sexual- and reproductive health and rights (SRHR), which in turn lead to poor health outcomes [2,3].

Realising these rights is especially challenging for populations in low- and middle-income countries (LMICs), and even more so for females and rural ethnic groups [4–7]. In LMICs an estimated 220 million females of reproductive age have an unmet need for family planning [4,5]. Due to their developing bodies and a lack of maternal services and support, adolescents are at a five times greater risk of maternal mortality compared to women aged 20 to 24 [6–9]. Young maternal age also has consequences for the infant, namely leading to low birth weight, preterm birth, and neonatal mortality [10–12]. Furthermore, adolescents are at an increased risk for unsafe abortions, sexually transmitted infections (STIs), and social phenomena such as early marriage and gender-based violence (GBV) [5,13]. These issues are context-specific, as they relate to cultural norms, traditions, and values, and also greatly depend on SRH knowledge, attitudes, and practices of adolescents [10]. Respecting, protecting and fulfilling adolescent sexual and reproductive health (ASRH) transcends beyond the wellbeing of adolescents; it also affects future generations and society [5,13].

In Lao, PDR youth are particularly at risk as the country has the youngest population in Southeast Asia, high early marriage and maternal mortality rates, and the highest adolescent birth rates in the region [14–16]. These high rates reflect the disparities in wealth and health across the country [14]. In addition, these rates indicate the lack of access to and quality of maternal healthcare services; youth-friendly services are particularly scarce [4,11]. With 50 official ethnicities and more than 200 ethnic subgroups, it is especially important to consider the socio-cultural factors contributing to ASRH in Lao PDR [14,16].

Adolescents are largely neglected in research and policy-making and context-specific factors and how these factors influence ASRH in Lao PDR is not yet known [17,18]. Therefore, in 2016, the Noi Framework was developed to raise awareness about adolescent issues in Lao PDR, to track and visualise the progress of these issues within the 2030 agenda, and to ensure that adolescent girls do not get left behind [19,20]. It is an Adolescent Girl Situation Analysis (AGSA) Framework that was developed by the Lao Tropical and Public Health Institute (Lao TPHI), the United Nations Population Fund (UNFPA), and Plan International [19]. Noi represents all adolescent girls in Lao PDR and until 2030 Noi’s progress within the SDGs will be checked annually [19]. This study utilises the Noi Framework and different elements from the socio-ecological model (SEM) and the social determinants of health (SDH) model to provide a more comprehensive, in-depth explanation of the factors that influence the SRH knowledge, attitudes, and practices of adolescents in Bokeo Province, Lao PDR. The information and insights gained from this study will add to the current efforts made to put ASRH on the Sustainable Development Agenda and to improving ASRH in Lao PDR.

## Methods

This study applied an analytical cross-sectional study design. Both quantitative and qualitative data were collected using multistage cluster sampling. Firstly, Bokeo Province was selected due to being ethnically diverse, with adolescent girls exposed various factors relating to the risk of being pregnant. Secondly, Paktha, Meung, and PhaOudom districts were selected because of poverty, limited infrastructure, and availability of healthcare services, and ethnic diversity. Finally, five villages, two in urban/semi-urban areas [villages within 10 km of the district capital], and three in rural/remote areas [villages more than 10 km from the district capital] were randomly selected in each district. There are numerous factors that affect SRH issues, namely poor socio-economic, socio-cultural, and environmental conditions, and inadequate accessibility, availability, and quality of SRH services [20]. Therefore, data on various demographic factors, SRH, other health-related topics, and gender roles were collected.

Bokeo Province is a small, mountainous area located in northwest Lao PDR, one of the most ethnically diverse, with one of the highest adolescent birth rates (113 births per 1,000 girls aged 15 to 19) [21,22] in the country. Face-to-face interviews were conducted with 837 adolescents using standardised questionnaires. Additionally, 59 key informant interviews (KIIs) were held with village chiefs, health workers, school directors, and teachers. Also, KIIs were conducted with 24 out-of-school and 17 in-school adolescents.

The questionnaire included nine demographic factors as independent variables (age, sex, ethnicity, religion, educational attainment, literacy level, employment status, district, and marital status), to explore their influence on SRH knowledge, attitudes, and practices as dependent variables. Variables are listed in Table 1.

**Table 1.** List of dependent and independent variables.

Dependent variables	Values	Type of variable
A. Knowledge of modern contraceptive method(s)	(0) No knowledge: *know no modern contraceptives* (1) Low knowledge: *know either the male condom or the pill* (2) Moderate knowledge: know the male condom and the pill* (3) High knowledge*: know the male condom, the pill, and one or more other modern contraceptives*	Ordinal
B. Autonomy	(0) Do not feel they have autonomy: *score of 0 to 1*^a^ (1) Partially feel they have autonomy: *score of 2 to 3* (2) Feel they have autonomy: *score of 4*	Ordinal
C. GBV towards females	(0) Do not accept GBV towards females^a^ (1) Accept GBV towards females	Dichotomous
D. GBV towards males	(0) Do not accept GBV towards males^a^ (1) Accept GBV towards males	Dichotomous
E. Sexually active	(0) Not sexually active^a^ (1) Sexually active	Dichotomous
F. Current contraception use	(0) Currently not using contraception^a^ (1) Currently using contraception	Dichotomous
Independent variables	**Values**	**Type of variable**
Age	10–19 years olds	Continuous
Sex	Female – Male^a^	Dichotomous
Marital status	Single^a^ – Married/in union^c^	Dichotomous
District	Paktha* – Pha Oudom – Meung	Nominal
Ethnicity^b^	Chinese-Tibetan^a^ – Mon-Khmer – Lao-Tai – Hmong-Mien	Nominal
Religion	Animism^a^ – Buddhism – Other/none	Nominal
Educational attainment	Primary education – Lower secondary education^a^ – Upper secondary education – Secondary education not defined – Never attended school	Ordinal
Literacy level	Illiterate – Partially literate^a^ – Literate	Ordinal
Employment status	Unemployed^a^ – Employed	Dichotomous

^a^reference category ^b^excluded after testing for multicollinearity ^c^in union refers to couples who are in a relationship, they may or may not live together, but are not married GBV = gender-based violence

Informants for KIIs were purposely selected and interviewed using semi-structured guidelines to gain in-depth knowledge about their views on, and experiences with school dropout, early marriage, adolescent pregnancy, gender equality, and gender roles. Audio recordings and field notes were made during the interviews. Transcripts were translated from Lao to English and paraphrased where necessary. Researchers from Lao TPHI and UNFPA did a thematic content analysis and made summaries of the transcripts with relevant codes. Etic and emic perspectives were used, and desk research was also incorporated.

Descriptive statistics were used to describe the demographic characteristics of the study population. Binary and multinomial logistic regression analyses were used to identify which independent variables were significant predictors of the dependent variables [23]. A p-value of less than 0.05 was considered statistically significant. Un-standardised regression coefficients (B), standardised regression coefficients (β), and odds ratios (ORs) were used to predict the direction and strength of the associations. To assess the precision of the prediction, 95% confidence intervals (CIs) for the β were used. The quality and goodness of fit of the models were determined using the Nagelkerke R-square, the overall accuracy rate, and for binary regression the Hosmer-Lemeshow (HL) test (HL value >0.05 indicating an improved fit of the model over the null model) was also used. A Chi-Squared test of independence was used to determine if there were relationships between the dependent variables. A p-value of less than 0.05 indicated significant associations, implying that the relative proportion of one variable is dependent on the other. Cramer’s V was used to measure the strength of the association (0 = no association, ≤0.2 = weak association, 0.2 to 0.3 = moderate association, and ≥0.3 = strong association) [24]. All statistical analyses were carried out in IBM SPSS Statistics version 25 [25].

The qualitative data were used to make sense of, and triangulate the quantitative findings, as well as to identify areas not covered in the questionnaires [26]. To present the findings several quotes were selected and paraphrased where necessary to increase their understandability.

The AGSA study obtained ethical approval from the National Ethics Committee for Health Research in Lao PDR. All respondents participated voluntarily and anonymously and prior to participating minor respondents had to give written assent together with written consent from a parent or guardian.

## Results

### Population characteristics

The mean age of the questionnaire respondents was 13.9 years (SD: 2.5) and 54.2% were girls. Further demographic characteristics of the study population are shown in Table 2. Ethnic distribution varied greatly across districts; Paktha had a predominant Lao-Tai population, Pha Oudum mostly Mon-Khmer’s, and in Meung the majority was Chinese-Tibetan. All Chinese-Tibetans lived in Meung and all Hmong-Miens lived in Paktha. Likewise, almost all Lao-Tais (95.6%) were Buddhists, and 95.1% of Chinese-Tibetan’s and 96.8% of Mon-Khmer’s were Animists.

**Table 2.** Demographic characteristics of the study population.

Characteristic	n (%)
**Age in five groups (n = 837)**	
10–11 years	180 (21.5)
12–13 years	205 (24.5)
14–15 years	225 (26.9)
16–17 years	145 (17.3)
18–19 years	82 (9.8)
**Sex (n = 837)**	
Female	454 (54.2)
Male	383 (45.8)
**Marital status (n = 835)**	
Single	791 (94.5)
Married/in union	44 (5.3)
**District (n = 837)**	
Paktha	289 (34.5)
Pha Oudom	260 (31.1)
Meung	288 (34.4)
**Religion (n = 828)**	
Animist	595 (71.1)
Buddhist	281 (26.0)
Christian/other/none	15 (1.8)
**Ethnicity (n = 802)**	
Chinese-Tibetan	270 (32.3)
Mon-Khmer	254 (30.3)
Lao-Tai	211 (25.2)
Hmong-Mien	67 (8.0)
**Educational attainment (n = 823)**	
Primary education	299 (35.7)
Lower secondary education	378 (45.2)
Upper secondary education	84 (10.0)
Secondary education not defined	31 (3.7)
Never attended school	31 (3.7)
**Literacy level (n = 770)**	
Illiterate	112 (14.5)
Partially literate	435 (56.5)
Literate	223 (29.0)
**Employment status (n = 833)**	
Unemployed	651 (78.2)
Employed	182 (21.8)

### Knowledge

Detailed findings on knowledge and practice are shown in Table 3. On average adolescents knew of 1.3 (SD: 1.1) of eight modern contraceptives, the most known was the male condom and the pill (60.2% and 51.5%, respectively). Approximately a third (32.4%) of the adolescents knew of no modern contraceptives, 15.6% knew either the male condom or the pill, the majority (33.0%) knew both these methods, and less than a fifth (19.1%) knew the male condom, the pill, and at least one other modern contraceptive. Although more boys knew either the male condom or the pill (19.7% of boys, compared to 12.2% of girls), knowledge levels did not vary significantly between the sexes. The main source for SRH information were schools, other mentioned sources were friends, siblings, doctors, media, and the radio.

**Table 3.** Final model of the significant predictors of knowledge and practices.

	B	SE	P-value	β	95% CI		
					Lower	Upper	
NO KNOWLEDGE (n = 579)							
**Intercept**	−0.927	1.074	0.388				
**Employment status (employed)**	0.639	0.289	0.027	1.894	1.075		3.338
**District: Pha Oudum ^(a)^**	1.754	0.336	0.000	5.778	2.991		11.163
**District: Meung ^(a)^**	1.178	0.320	0.000	3.247	1.733		6.085
**Religion: Buddhist ^(b)^**	−0.910	0.348	0.009	0.402	0.203		0.797
LOW KNOWLEDGE (n = 579)							
**Intercept**	−3.732	1.303	8.204				
**Sex (male)**	0.723	0.271	0.008	2.060	1.210		3.506
HIGH KNOWLEDGE (n = 579)							
**Intercept**	−2.705	1.381	0.050				
**Age**	0.171	0.082	0.038	1.186	1.010		1.394
**District: Pha Oudum ^(a)^**	1.647	0.342	0.000	5.190	2.653		10.154
**Educational attainment: Upper secondary education ^(c)^**	1.090	0.441	0.013	2.974	1.253		7.057
^(a)^ compared to adolescents living in Paktha ^(b)^ compared to Animists ^(c)^ compared to adolescents with lower secondary education Reference category: Moderate knowledge							
SEXUALLY ACTIVE (n = 739)							
**Constant**	−19.818	1.899	0.000	0.000			
**Age**	0.631	0.092	0.000	1.879	1.569		2.250
**Sex (male)**	1.243	0.356	0.000	43.465	1.725		6.958
**Marital status (married/in union)**	3.438	0.539	0.000	31.110	10.809		89.539
**District**			0.000				
**District: Pha Oudum ^(a)^**	0.352	0.428	0.410	1.422	0.615		3.288
**District: Meung ^(a)^**	1.710	0.425	0.000	5.527	2.401		12.725
**Employment status (employed)**	1.107	0.323	0.001	3.024	1.605		5.700
^(a)^ compared to adolescents living in Paktha Reference category: Not sexually active							
CURRENT CONTRACEPTION USE (n = 575)							
**Constant**	−12.879	1.829	0.000	0.000			
**Age**	0.635	0.108	0.000	1.887	1.527		2.331
Reference category: Currently not using contraception							

Misconceptions about modern contraceptives existed; almost half (48.3%) thought the pill prevents HIV and other STIs, of which the majority (83.7%) had been taught about the pill in school, and almost a third (29.1%) also thought the pill did not prevent pregnancy. Additionally, one-fifth (20.7%) stated male condoms do not prevent HIV/STIs, and one-sixth (15.5%) stated condoms do not prevent pregnancy.

The significant predictors of knowledge of modern contraceptives were age, educational attainment, sex, religion, employment status, and district of residence. For every additional year in age, adolescents were 1.19 times more likely to have higher knowledge. Adolescents with upper secondary education were also more likely to have high knowledge (β: 2.97). Boys were more likely to have low knowledge (β: 2.06). Buddhists and employed adolescents were less likely to have no knowledge, compared to Animists and unemployed adolescents (β: 0.40 and β: 1.89, respectively). The most prominent predictor was district; Pha Oudum and Meung had a significantly increased odds of having no knowledge, compared to Paktha residents (β: 5.78 and β: 3.25, respectively). Pha Oudum residents were also more likely to have high knowledge (β: 5.19). Marital status and literacy level were not significant predictors. The predictors explained roughly 35.7% of the variation in knowledge and the overall accuracy rate was 52.2% in terms of predicting which adolescents fell into different knowledge categories.

### Practices

Ninety-one adolescents, including those married and in union, reported having had sex, 38 girls (8%) and 53 boys (14%). Just over a third (35.2%) of them currently used contraception. Of the sexually active adolescents, 87.8% considered themselves as having sexual autonomy and, of the adolescents using contraception, 80.6% expressed that they were autonomous regarding contraceptive use. Condoms and the pill were most used, followed by the traditional withdrawal method (63.9%, 22.2%, and 8.3%, respectively).

The odds of being sexually active were much higher for boys (β: 43.5) and for married/in union adolescents (β: 31.1). The odds of being sexually active also increased with every additional year in age, for adolescents who were employed, and for Pha Oudum and Meung residents (β: 1.9, β: 3.0, β: 1.4, β: 5.5). The predictors explained roughly 57.1% of the variation in practices and the model was a good fit (HL value: 0.93). It was better at predicting who not had sex (98.2%) than who had (56.0%). For every additional year in age, adolescents were 1.9 times more likely to currently be using contraception. Age explained roughly 20.7% of the variation in knowledge and the model was a good fit (HL value: 0.19). The model could accurately predict who did not use contraception, but not who did.

### Attitudes – autonomy

Detailed findings on autonomy and GBV are shown in Table 4. More than a quarter (27.5%) of the adolescents felt they did not have autonomy regarding sex, contraception use, having a baby, and marriage, 19.1% felt they partially had autonomy, and more than half (53.4%) felt they did have autonomy. Sexual autonomy was highest (71.1%) and autonomy regarding marriage was lowest, but almost 60% indicated that they were autonomous regarding their choice in marriage. This also came forward in the interviews, where married boys and girls reported having autonomy regarding marriage.

I decided myself (to get married) because I love him. (Adolescent girl)

**Table 4.** Final model of the significant predictors of autonomy and GBV.

	B	SE	P-value	β	95% CI		
					Lower		Upper
DO NOT FEEL THEY HAVE AUTONOMY (n = 551)							
**Intercept**	2.992	1.127	0.008				
**Age**	−0.293	0.075	0.000	0.746	0.644	0.864	
**District: Pha Oudum ^(a)^**	1.858	0.345	0.000	6.411	3.263	12.594	
**District: Meung ^(a)^**	1.666	0.339	0.000	5.292	2.721	10.292	
**Religion: Christianity/other/none ^(b)^**	2.690	1.179	0.023	14.726	1.461	148.403	
**Educational attainment: Primary education ^(c)^**	0.706	0.279	0.011	2.025	1.172	3.498	
DO NOT FEEL THEY HAVE AUTONOMY (n = 551)							
**Intercept**	2.992	1.127	0.008				
**Age**	−0.293	0.075	0.000	0.746	0.644	0.864	
**District: Pha Oudum ^(a)^**	1.858	0.345	0.000	6.411	3.263	12.594	
**District: Meung ^(a)^**	1.666	0.339	0.000	5.292	2.721	10.292	
**Religion: Christianity/other/none ^(b)^**	2.690	1.179	0.023	14.726	1.461	148.403	
**Educational attainment: Primary education ^(c)^**	0.706	0.279	0.011	2.025	1.172	3.498	
PARTIALLY FEEL THEY HAVE AUTONOMY (n = 551)							
**Intercept**	−1.599	1.101	0.147				
**Marital status (married/in union)**	1.090	0.431	0.012	2.974	1.277	6.927	
**District: Pha Oudum ^(a)^**	1.330	0.306	0.000	3.782	2.078	6.885	
**Religion: Christianity/other/none ^(b)^**	2.441	1.192	0.041	11.481	1.109	118.812	
^(a)^ compared to adolescents living in Paktha ^(b)^ compared to Animists ^(c)^ compared to adolescents with lower secondary education Reference category: Do feel they have autonomy							
ACCEPT GBV TOWARDS FEMALES (n = 717)							
**Constant**	−2.280	0.491	0.000	0.102			
**Sex (male)**	0.347	0.170	0.042	1.415	1.013	1.976	
**Marital status (married/in union)**	0.856	0.357	0.017	2.354	1.169	4.742	
**District**			0.000				
**District: Pha Oudum ^(a)^**	0.385	0.193	0.046	1.469	1.007	2.143	
**District: Meung ^(a)^**	−0.728	0.227	0.001	0.483	0.309	0.754	
^(a)^ compared to adolescents living in Paktha Reference category: Do not accept GBV towards females GBV = gender-based violence							
ACCEPT GBV TOWARDS MALES (n = 727)							
**Constant**	−2.615	0.510	0.000	0.000			
**Sex (male)**	0.481	0.176	0.006	1.618	1.145	2.287	
**Marital status (married/in union)**	0.770	0.366	0.035	2.161	1.055	4.423	
**District**			0.003				
**District: Pha Oudum ^(a)^**	0.283	0.201	0.159	1.327	0.895	1.967	
**District: Meung ^(a)^**	−0.527	0.232	0.023	0.591	0.375	0.931	

^(a)^compared to adolescents living in Paktha

Reference category: Do not accept GBV towards males

GBV = gender-based violence

However, marriage – and being a parent – deprived them from their autonomy to go to school. The adolescent in the following quote recognised this as a disadvantage of early marriage.

I am happy that I have a husband, but at the same time I am not happy because I could not study.” – Adolescent girl (19 years old, out-of-school, married, 7 months pregnant, Houaypha village in Paktha district)

The significant predictors of autonomy were age, educational attainment, district, marital status, and religion. For every additional year in age, adolescents were 0.75 times less likely to feel like they did not have autonomy. Adolescents with primary education and Pha Oudum and Meung residents were more likely to feel like they did not have autonomy (β: 2.0, β: 6.4, and β: 5.3, respectively). Pha Oudum residents and married/in union adolescents were more likely to partially have autonomy (β: 3.8 and β: 3.0). The confidence intervals for religion (Christian/other/none) were wide, so these results were not interpreted. The predictors explained 24.9% of the variation in autonomy. The overall accuracy rate was 60.3% in terms of predicting which adolescents fell into the different autonomy categories.

### Attitudes – gender equality

The attitudes towards gender equality were conflicting. The majority (93.5%) believed that girls and boys should have the same opportunities to attend school, yet 73.1% of these adolescents also believed that pregnant girls should not be allowed to attend school. This contradictory perspective on gender equality was substantiated in the interviews, where most key informants stated that girls and boys have equal opportunities, yet many still viewed males as the best suited to be head of household.

At school, girls and boys are equal. Now woman can be a leader and a village chief. Head of family should be a man. – Village chief (Pha Oudum district)

In regard to GBV, approximately a quarter stated there were times when a female deserves to be disciplined or beaten by a male (27.9%), and vice versa (24.8%). In a domestic setting, neglecting children was the situation in which violence was most justified (55.9% justified against the wife and 47.1% against the husband). In three of the four situations, hitting or beating the wife was considered more justified than hitting or beating the husband.

The significant predictors of GBV towards females and males were the same, namely: sex, marital status, and district. Boys were 1.4 and 1.6 times more likely to accept GBV toward females and males, respectively. Married/in union adolescents were 2.4 and 2.2 times more likely, and Pha Oudum residents were 1.5 and 1.3 times more likely to accept GBV towards females and males, respectively. Meung residents were less likely to accept GBV towards females and males (β: 0.48 and β: 0.59, respectively). The models were a good fit (HL value: 0.40 for GBV towards females and 0.06 for GBV towards males); yet the predictors only explained roughly 4.4% and 3.0% of the variation in the respective variables. The overall accuracy rate was 71.1% for GBV towards females and 75.2% for GBV towards males in terms of predicting which adolescents fall into different categories.

### Relationships between dependent variables

There was no significant association between knowledge and sexual activity, or between knowledge and GBV towards females. However, there was a weak association between knowledge and current contraception use (Cramer’s v: 0.18), and between knowledge and GBV towards males (Cramer’s v: 0.13). Noteworthy was the strong positive association between knowledge and autonomy (Cramer’s v: 0.36). Most of the adolescents who felt they did not have autonomy also had little knowledge, whilst adolescents that felt they did have autonomy more often had moderate or high knowledge. Additionally, there was a weak association between autonomy and sexual activity (Cramer’s v: 0.13), and between autonomy and current contraception use (Cramer’s v: 0.12). Lastly, there was a moderate association between autonomy and GBV towards females and males (Cramer’s v: 0.26 and Cramer’s v: 0.24, respectively).

## Discussion

This study illustrates that biological, residential, socio-economic, and socio-cultural factors play an important role in ASRH. Additionally, rural and non-Lao-Tai ethnic groups were often negatively associated with SRH outcomes. Three main areas requiring more effort and greater investment for ASRH have been identified: poor knowledge and use of contraceptives, gender inequality, and lack of autonomy regarding SRH-related decisions. Especially the latter two have yet to be depicted comprehensively in literature about Lao PDR.

We have reported a prevalence of sexual activity of 11%. This is lower than most recent studies among adolescents that presented a varying prevalence of sexual activity among adolescents ranging from 14% to 56% of those aged 16 to 19 years being sexually experienced [4,27–29]. However, these studies did not include adolescents aged 11–15, who are less sexually active. Similarly to other studies conducted in Lao PDR, the level of unsafe premarital sex was quite high and knowledge of modern contraceptives was low. Low knowledge was predicted by poor education, young age, living in rural areas and belonging to a small ethnic group; on average adolescents knew one of the eight methods, almost a third knew no methods, and misconceptions existed [8,14,30–32]. Contraceptive use was only associated with knowledge to a limited extent. Also, the association between autonomy and contraceptive use in this study was weak, whereas previous studies in Asia have found autonomy to be of great influence on contraceptive use [33,34]. We can confirm that factors such as knowledge and autonomy contribute to contraceptive use, but they all are just pieces of the overall puzzle, in that the use of contraceptives is determined by more than knowledge and autonomy, and may be either related to an individual (e.g. attitudes) and their environment (e.g. access and acceptability). Thus, we confirm international literature on the multifactorial nature of contraceptive use [35]. Nevertheless, it remains important that knowledge gaps and misconceptions about contraceptives and their side effects must be provided and the autonomy of girls needs to be strengthened, but interventions targeting an isolated factor will not suffice.

Attitudes towards gender equality and GBV were ambiguous. At first glance, perspectives seemed to be supportive of equal opportunities, but further questioning about specific situations and roles indicated otherwise. Females were not seen as suitable to be heads of households and it was not considered appropriate for pregnant girls to attend school. Moreover, this study and others in Lao PDR found boys’ education to be prioritised, which is likely because boys generally stay at home, whilst girls leave home after they get married [8,36,37]. Similarly, the majority of adolescents said they were against GBV, but in a domestic setting, and particularly against the wife, violence was widely accepted. This is consistent with literature that states that GBV predominantly affects females [38,39].

### Practical implications and further research

Our findings indicate the need to protect and concentrate efforts on girls, particularly in rural districts. Gender inequality and discrimination increase chances of early marriage, deprive adolescents of basic human rights, and hinder their future prospects [13,40]. For this reason, the life-skills curriculum and comprehensive sexuality education (CSE) need to be further integrated into the national core curriculum in Lao PDR. CSE focuses on gender equality, participation, consent, and empowerment, which are all vital for establishing equal opportunities, positive gender attitudes, and autonomy [14,41,42].

The main SRH information source was school, and since educating out-of-school adolescents poses greater challenges, the focus must be on keeping adolescents in school as well as reintegration of drop-out adolescents into schools [18,43]. However, the problem in Lao PDR is that adolescents often drop out before secondary school, and so they do not receive comprehensive sexuality education, which is currently taught from Grade 6 of secondary school [4,8]. Moreover, this study, as well as other studies conducted in Lao PDR and other southeast Asian countries, found teachers to be inadequately trained and equipped to provide quality CSE [15,43–45]. Therefore, investment also has to be put into training and equipping teachers with the resources they need.

Nevertheless, teachers’ own cultural norms and values might make them hesitant to teach CSE effectively, or, as expressed by several key informants, they might think that teaching adolescents CSE is inappropriate [41]. The latter finding suggests that although the poor current teaching quality was largely recognised, not everyone was ready or open to changing the situation.

Lastly, it is important to note that we found the having knowledge did not imply that adolescents knew how to use or obtain the contraceptives they needed. Merely knowing about contraceptive method is not sufficient; studies have shown that adolescents with basic sexual health knowledge rarely put their knowledge into practice and seldom utilise health services. Therefore, it is important that investment goes into youth-friendly and accessible SRH services [43], especially since adolescents often fear stigma or embarrassment when accessing these services, and most healthcare providers lack specific training in helping adolescents [8].

The findings from the AGSA are being used for developing the national youth and adolescent health policy and programming for adolescents sexual and reproductive health, comprehensive sexuality education in schools and expanding the coverage of youth-friendly services. This study also provides valuable insights into research questions raised in the National Research Agenda of Lao PDR under the domain of sexual health and the avenues of sexual health education and unintended pregnancies [46].

## Strengths and limitations

The cross-sectional study design meant that correlation, but not causation could be determined. Nevertheless, numerous predictors were examined to obtain a comprehensive picture of the factors that contribute to SRH knowledge, attitudes, and practices, and a high Cronbach’s alpha indicated good internal consistency and reliability.

We have not reported extensively on our qualitative findings, yet these largely support our quantitative findings, and provided depth to our findings, especially where quantitative data were inconclusive with respect to gender equality and GBV. However, our study did not touch upon many structural factors that span the socio-ecological domain that contribute to the use of contraceptives, such as easy access.

Despite extensive training of interviewers, this study might have suffered from a common challenge with research into SRH and adolescents, taboos and socially desirable answers. For example, adolescents were given the option to skip questions. This could have led to an underreporting of attitudes and practices [47,48]. Additionally, data were collected via face-to-face interviews, and so adolescents might have given socially desirable answers or answered ‘don’t know’.

## Conclusion

This study illustrates the poor knowledge on contraceptive methods and helps in understanding the views and perceptions of adolescents and key informants on gender equality and GBV. The authors emphasise the need to further integrate CSE, to introduce it in primary school, and to increase investment in training and monitoring teachers. Given the evident link found in this study, as well as in previous studies, between education and increased SRH knowledge, autonomy, and safer sexual practices, it is vital that policies and programmes focus on keeping adolescents in school. Better understanding of gender-based violence should be developed and gender-sensitive attitudes, behaviours, and power dynamics should be promoted among adolescents and in their communities.

## Paper context

The Lao PDR MoH and the UNFPA in Lao PDR have recognised the gap in research and policies specifically addressing youth. Therefore, this study was conducted. The findings from the AGSA are being used for developing the provincial level adolescent’s girl profile in Lao PDR, national youth and adolescent health policy and programming for adolescents sexual and reproductive health, comprehensive sexuality education in schools and expanding the coverage of youth-friendly services.
